# Upper-Bound Energy Minimization to Search for Stable Functional Materials with Graph Neural Networks

**DOI:** 10.1021/jacsau.2c00540

**Published:** 2022-12-31

**Authors:** Jeffrey N. Law, Shubham Pandey, Prashun Gorai, Peter C. St. John

**Affiliations:** † Biosciences Center, National Renewable Energy Laboratory, Golden, Colorado80401, United States; ‡ Department of Metallurgical and Materials Engineering, Colorado School of Mines, Golden, Colorado80401, United States; § Materials Science Center, National Renewable Energy Laboratory, Golden, Colorado80401, United States

**Keywords:** materials discovery, structure prediction, graph neural networks, solid state batteries, reinforcement learning

## Abstract

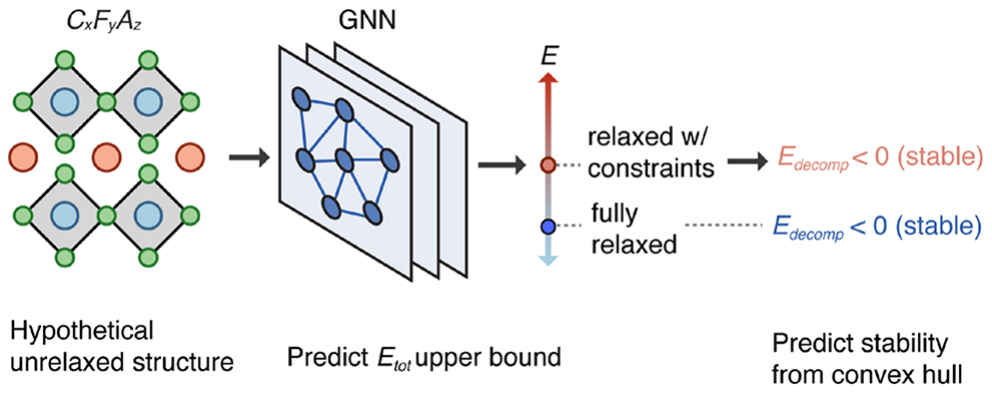

The discovery of new materials in unexplored chemical spaces necessitates quick and accurate prediction of thermodynamic stability, often assessed using density functional theory (DFT), and efficient search strategies. Here, we develop a new approach to finding stable inorganic functional materials. We start by defining an upper bound to the fully relaxed energy obtained via DFT as the energy resulting from a constrained optimization over only cell volume. Because the fractional atomic coordinates for these calculations are known *a priori*, this upper bound energy can be quickly and accurately predicted with a scale-invariant graph neural network (GNN). We generate new structures via ionic substitution of known prototypes, and train our GNN on a new database of 128 000 DFT calculations comprising both fully relaxed and volume-only relaxed structures. By minimizing the predicted upper-bound energy, we discover new stable structures with over 99% accuracy (versus DFT). We demonstrate the method by finding promising new candidates for solid-state battery (SSB) electrolytes that not only possess the required stability, but also additional functional properties such as large electrochemical stability windows and high conduction ion fraction. We expect this proposed framework to be directly applicable to a wide range of design challenges in materials science.

## 1 Introduction

Overcoming critical barriers in materials science will require the discovery of yet unknown structures with precisely tailored properties. Computational searches driven by quantum chemistry calculations have accelerated materials exploration over large compositional and structural spaces, but are still limited to few tens of thousands of materials.^1−4^ Often, such searches are restricted to structures previously documented in crystallographic databases such as the Inorganic Crystal Structure Database (ICSD)^5^ and Cambridge Structural Database (CSD),^6^ which predominantly comprise experimentally synthesized inorganic materials. Their use in discovery campaigns for novel materials is therefore limited. Solid-state batteries (SSBs) are one such application, where the use of solid electrolytes makes SSBs safer and more energy dense than traditional Li-ion technology. SSBs require materials that meet several performance metrics,^7^ and remain limited by the thermodynamic instability of electrode–electrolyte interfaces.^8,9^ For instance, well-known solid electrolytes such as Li_10_GeP_2_S_12_, Li_6_PS_5_Cl (argyrodite), and Li_7_La_3_Zr_2_O_12_ decompose at the interface with Li-metal anode forming solid-electrolyte interphases that are difficult to control and cause performance degradation.^10,11^ Therefore, further improvements to SSB design require searching for new materials that are stable at suitable reduction and oxidation potentials.

A central problem in using machine learning (ML) methods to accelerate the search for novel crystal structures is finding structures that are thermodynamically stable, i.e., stable against decomposition into competing phases. Compositional models are not sufficiently accurate to reliably predict thermodynamic stability.^12,13^ Graph neural network (GNN) models have achieved impressive results in predicting formation energy and decomposition energy with mean absolute error (MAE) close to chemical accuracy (0.03–0.05 eV/atom).^14−17^ However, GNN models require the crystal structure as inputs, which are available only after performing expensive density functional theory (DFT) relaxations. A considerably harder problem is predicting *a priori* whether a hypothetical crystal structure input will be stable before performing DFT relaxation. Recent studies have made some progress in this direction through the use of scale-invariant approaches, where the volume of input structures is normalized to make GNN models less sensitive to volume changes that often occur during relaxation.^18,19^ It has been previously shown that including high-energy structures as training data is critical to developing models intended to rank the stability of potential crystal structures for a given composition.^13^ Analogously, training GNN models only on fully relaxed structures and their DFT total energies may bias the models to under-predict the energies of high-energy structures in unfavorable arrangements.

In this work, we develop a generalized approach for finding new inorganic crystal structures that are likely to be stable. We first demonstrate that the success of scale-invariant approaches in predicting the DFT-relaxed total energy of a crystal structure depends heavily on the degree to which the structure relaxes away from the initial unrelaxed structure. In a wide search over structures created by ionic substitution,^21^ DFT often alters the initial structures drastically during relaxation to a local energy minimum. Predictive models for energy trained with these unrelaxed structures as inputs are therefore inaccurate and unsuitable for screening potentially stable decorations.

We present an alternate approach to finding new stable structures over a large decoration space that is compositionally and structurally diverse. First, we construct a database of constrained DFT relaxations over only the unit cell volume, which by design yields an upper bound to the total energy of the unconstrained (full) relaxation. This upper-bound energy can be predicted to a high accuracy by scale-invariant ML models (MAE ∼ 0.05 eV/atom), since fractional coordinates in the unit cell are known precisely. By subsequently searching for decorations that minimize this predicted upper-bound energy, we find novel crystal structures that are highly likely to be stable. Out of 14.3 million decorated structures, this approach predicted a stable structure for 2003 compositions. Validating these top candidates with DFT confirmed >99% of them to be thermodynamically stable, *i.e.*, having negative decomposition energy. We find many of these stable candidates also have suitable functional properties for SSBs, with structural similarities to previously explored solid electrolytes, electrodes, and coatings.

Expanding our search to an even larger number of compositions and prototypes will exponentially increase the search space, making it computationally intractable to exhaustively assess the stability of every structure. To address this future need, we demonstrate a reinforcement learning (RL)-augmented search strategy that finds stable structures using our surrogate stability function at a fraction of the computational cost. Overall, this study shows that ML strategies are able to drastically reduce the computational cost and time to find promising inorganic functional materials.

## 2 Results and Discussion

### 2.1 Challenges in Predicting Thermodynamic Stability

To be useful in screening candidate structures for stability, a machine learning (ML) surrogate model must be able to predict the total energy of a relaxed structure using only information available before the relaxation is performed. To provide our surrogate model with relevant training examples, we first constructed a database of example hypothetical structures through ionic substitution with compositions suitable for SSBs. We selected 67 489 candidate structures for full DFT relaxation by decorating prototype ICSD structures with new compositions (Section 4.3.2). We refer to this as the *fully relaxed* data set. The corresponding total energies in this data set are denoted by *E*_*tot*_.

**Figure 1:**
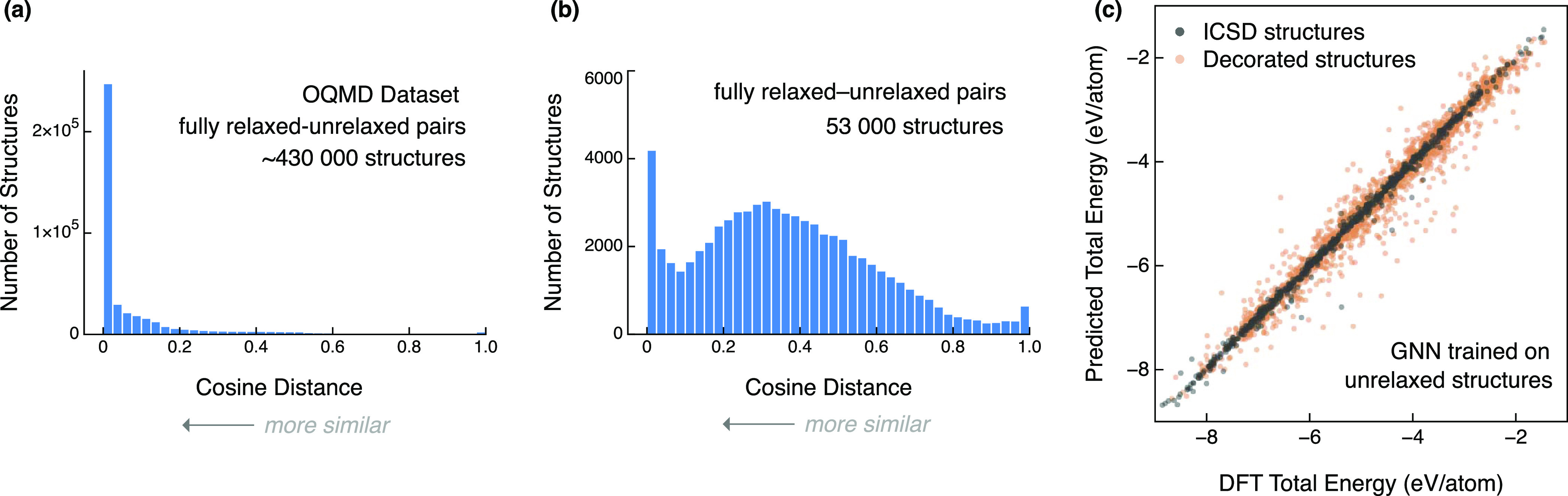
Initial surrogate model development. (a) Cosine distances between initial and DFT-relaxed structures in the Open Quantum Materials Database (OQMD).^18,20^ (b) Cosine distances between initial and DFT-relaxed structures for the 53 000 hypothetical decorated structures used in this study (Figure 2). (c) Predicted vs DFT total energy of a GNN trained on ICSD and *unrelaxed* hypothetical structures and evaluated on the energy of the DFT-relaxed structures. The prediction accuracy for ICSD structures (gray) is high, but low for unrelaxed hypothetical structures (orange).

We first trained a scale-invariant GNN model (Section 4.1) on the ICSD and fully relaxed data sets, where we paired the unrelaxed structures with their corresponding total energy after relaxation. We withheld ∼5% of structures in each data set for the validation and test sets. While the model performed well on ICSD structures (gray points in Figure 1c, MAE = 0.05 eV/atom), we found that the error was much larger on the fully relaxed data set (orange points in Figure 1c, MAE = 0.13 eV/atom). The higher MAE for the fully relaxed data set is likely due to many of the input structures starting in highly unfavorable configurations, such that DFT relaxation significantly alters their volume, cell shape geometry, and fractional atomic coordinates in finding a local energy minimum.

To quantify the structural change during DFT relaxation, we computed the cosine distance between the unrelaxed and relaxed structure pairs using Matminer fingerprints (see Section 4.4).^22^ Here, a cosine distance of zero indicates high structural similarity, ignoring any volume changes. With our wide range of prototypes and decorations, we found that over 86% of pairs had a cosine distance above 0.1, meaning the vast majority of structures change quite dramatically after relaxation (Figure 1b). This is in stark contrast to the distribution of distances for structures in the Open Quantum Materials Database (OQMD)^20^ (Figure 1a), where similar GNN models are able to achieve high accuracy for unrelaxed structures, *i.e.*, MAE < 0.05 eV/atom.^18^ This result implies that while scale-invariant methods are robust to changes in the cell volume during DFT relaxation, they cannot account for large changes in fractional coordinates and cell shape geometry when DFT relaxes a structure far away from a high-energy starting configuration.

### 2.2 Volume-Only Structure Relaxations

Rather than attempt to directly predict the energy resulting from a full DFT relaxation, we developed an alternate approach. We noticed in our fully relaxed data set that when a prototype decoration was favorable for a new composition, the structure tends to relax with minimal changes in its fractional coordinates. A surrogate model with the goal of differentiating between favorable and unfavorable decorations would need examples of each to perform well. We therefore constructed a second database of DFT relaxed structures where we fix the unit cell geometry and fractional atomic coordinates and only relax their volumes. By design, the cosine distances between the unrelaxed and volume relaxed pairs are zero.

**Figure 2:**
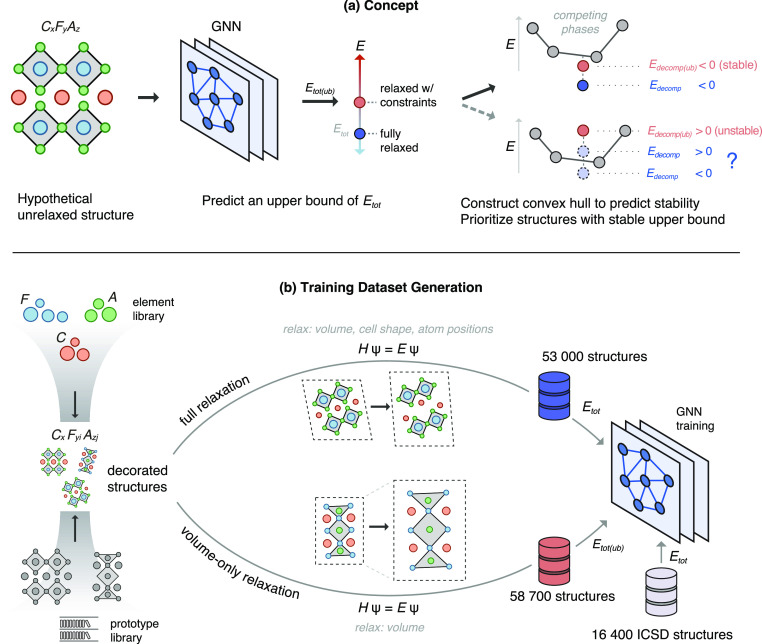
Overview of approach and data set generation. (a) Starting from an unrelaxed structure, we predict an upper bound for the total energy i.e., energy after a constrained relaxation. We then evaluate the thermodynamic stability relative to competing phases and prioritize structures predicted to be stable, meaning the upper bound of the decomposition energy is <0 eV/atom. If the decomposition energy upper bound is >0 eV/atom, the structure could still be stable after full relaxation. (b) Our element library consists of conducting ions (*C*), framework cations (*F*), and anions (*A*). We build valence-balanced compositions of the general form , where *x*, *y*_*i*_, and *z*_*j*_ are the stoichiometries corresponding to *C*, *F*, and *A*, respectively. Here, *i* and *j* are 1–2; *i.e.*, we consider up to 2 framework cations and 2 anions. For a given composition, we decorate the elements in prototype structures (from a prototype library) via ionic substitution. These structures are then relaxed with DFT in two ways: (i) full relaxation and (ii) volume-only relaxation, where the cell shape and atom positions are held constant.

The success of scale-invariant GNNs in previous applications suggests that the optimal volume and energy for a given structure can be predicted by its fractional coordinates and cell shape geometry.^18,19^ By constraining these features during a volume-only relaxation, we are able to augment our training set with high-energy examples, and provide a better foundation to distinguish favorable from unfavorable structure decorations. The volume relaxation also provides us with an accurate upper bound to the total energy calculated by the unconstrained relaxation, since the energy must stay the same or decrease when DFT is allowed to fully relax the structure. Note that this upper bound is not a theoretical limit to the total energy of the structure, but serves as a useful reference point. While searching for stable structures in a large decoration space, if a volume relaxed structure is not predicted to be stable w.r.t. competing phases, the fully relaxed structure may or may not be stable. However, if a volume relaxed structure is stable, the fully relaxed structure will be stable as well. Assuming at least some of the volume relaxed structures are stable, we can efficiently screen for them in the unrelaxed decoration space using an accurate surrogate model (Figure 2a).

**Figure 3:**
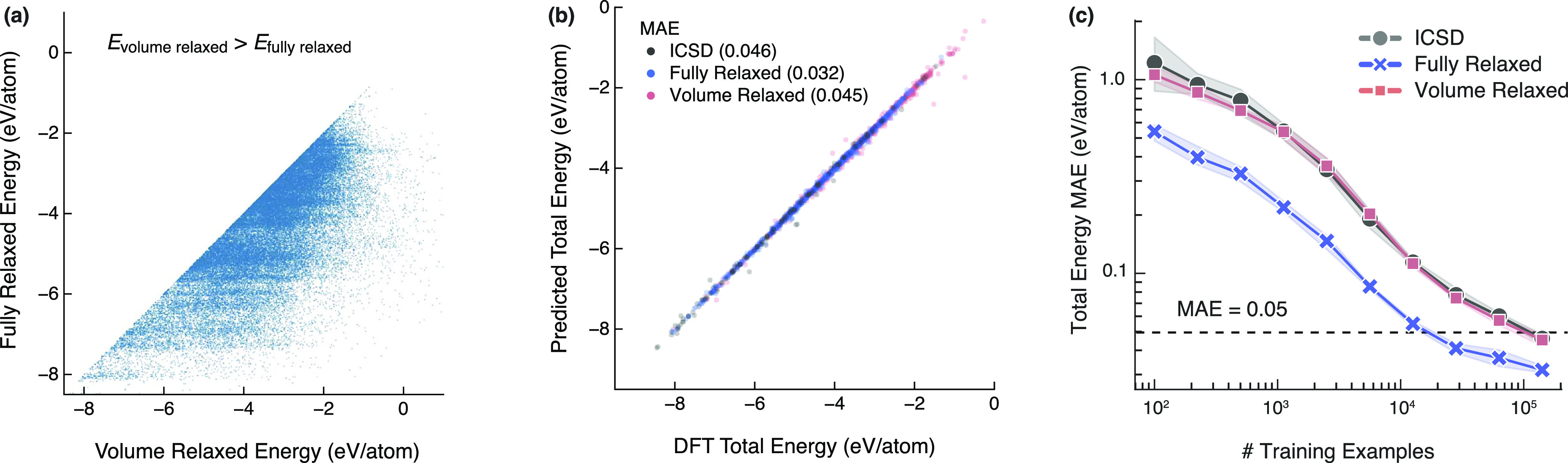
Effect of the volume relaxed data set on energy and surrogate model performance. (a) DFT energy differences between volume relaxed and fully relaxed structures. (b) Predicted vs DFT total energy of the model trained and evaluated on the three data sets. (c) Learning curves of the model’s prediction error by data set size.

We performed a volume-only relaxation on each of the ∼68 000 unrelaxed structures used as inputs to the fully relaxed data set. We pruned ∼9000 that did not pass quality control filters (Section 4.3.4) and refer to these ∼58 700 structures as the *volume relaxed* data set (Figure 2b). Figure 3a shows the differences in total energy between the two data sets, which confirms that the volume-only relaxations are indeed an upper bound. These volume-only relaxations augment the original fully relaxed data set, providing examples of high-energy decorations that will serve to guide an ML model toward choosing low-energy initial structures.

With the addition of the volume relaxed data set, we trained a scale-invariant GNN on all three data sets (Figure 2b). The model uses a scale-invariant approach, scaling the input crystal’s volume to make the minimum edge length 1 Å (section 4.1). In mixing the volume relaxed with the ICSD and fully relaxed data sets, we use the relaxed geometry for the fully relaxed structures instead of their input geometry. In this way, the model is tasked with predicting the total energy of a structure in its given, scale-invariant configuration, rather than attempt to predict the energy to which an input geometry might ultimately relax. The prediction accuracy for all structures improved substantially (Figure 3b), as the GNN had access to the correct fractional coordinates for all inputs. Learning curves of the prediction error as a function of the data set size show the model benefits from additional data up to the full data set size (Figure 3c).

### 2.3 Surrogate Model Predictions

With the trained GNN surrogate model, we predicted the upper bound energy of all 14.3 million possible decorations (Section 4.2). Because predicting a candidate structure’s upper-bound energy requires only a single forward pass through our trained ML model, evaluation of all 14.3 million took only 2 h using a single Tesla V100 GPU accelerator. We estimated the thermodynamic phase stability of each of these structures by computing their decomposition energy obtained through a convex hull analysis. Here, the convex hull is constructed by considering competing phases from the ICSD. The total energy of ICSD structures is taken from the NREL Materials Database,^23^ as explained in Section 4.3.3. The decomposition energy (*E*_decomp_) is a measure of the thermodynamic stability of a structure against chemical decomposition into competing phases.^12^*E*_decomp_ is the minimum energy that the formation energy of an unstable material has to be lowered (more negative) before it becomes stable. Similarly, for a stable compound, *E*_decomp_ is the maximum energy that the formation energy can be increased (less negative) before it becomes unstable.^12^ For each composition, we selected the structure with the lowest predicted energy. About 1.7% of compositions (3719) had a structure with a negative *E*_decomp_ < 0.001 eV/atom. To account for potential errors in the model predictions, we applied a more stringent cutoff of a negative *E*_decomp_ < −0.1 eV/atom, which resulted in 2003 compositions. Before analyzing these structures, we first validate the predicted stable structures with DFT.

### 2.4 DFT Confirmation of Predicted Stable Structures

**Figure 4:**
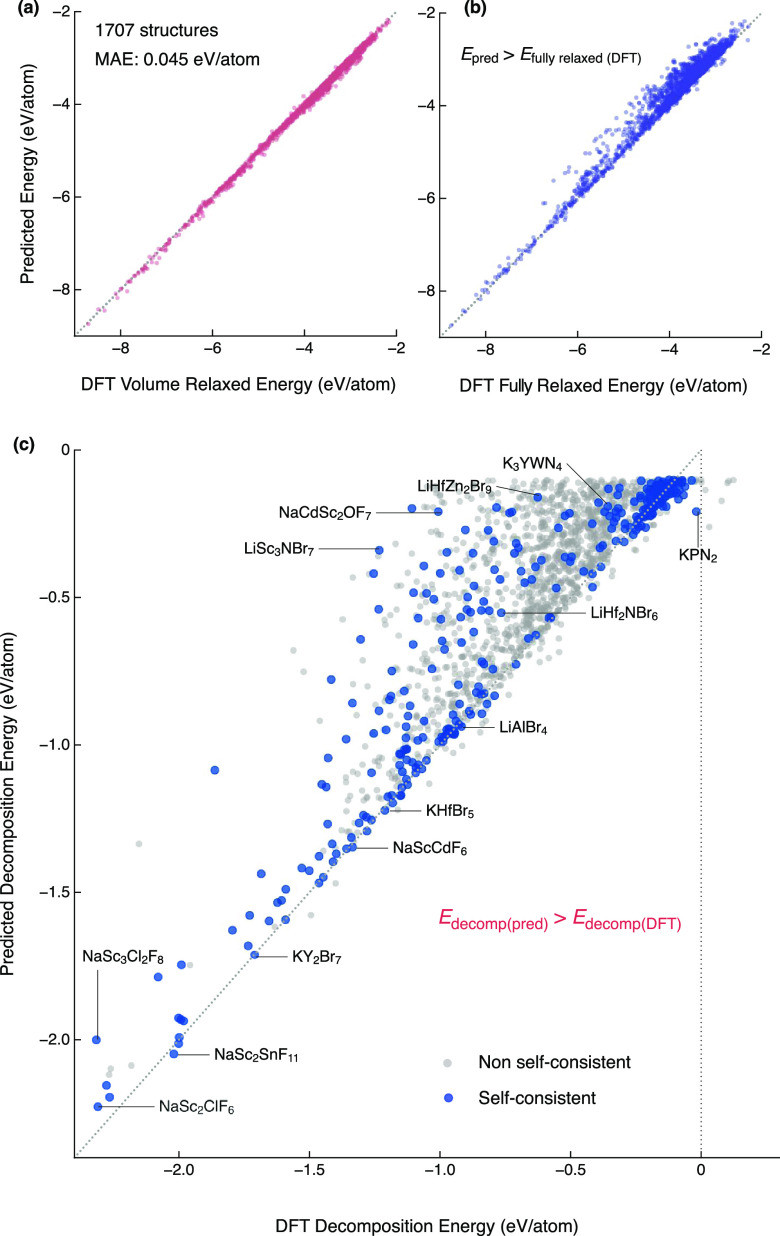
DFT confirmation of predicted stable structures. (a) Predicted vs volume-only DFT relaxation total energy. (b) Predicted vs full DFT relaxation total energy. (c) Predicted vs full DFT relaxation decomposition energy. Points in blue indicate structures that remain stable after evaluation of the self-consistent decomposition energy (Section 2.4).

We performed both full DFT relaxation and volume-only relaxation for the 2003 predicted stable structures. Each of these 2003 structures has a unique composition because we chose the lowest-energy structure for a given composition. Of the 1707 structures where the DFT calculations successfully converged, we find the model predicts the energy upper bound, *i.e.*, volume relaxed total energy, to a high accuracy (MAE = 0.045 eV/atom, Figure 4a). Nearly all predicted upper-bound total energies are larger than the fully relaxed DFT energies (Figure 4b), consistent with our hypothesis that the volume relaxed energies are an upper bound. We confirm that 99% (1700/1707) of the predicted structures are in fact stable, as determined from a convex hull analysis (Figure 4c). The DFT decomposition energies (calculated using fully relaxed DFT total energy) are more negative i.e., more stable, than the predicted values, demonstrating the success of our upper-bound approach.

*E*_decomp_ in Figure 4c are calculated from a convex hull construction by considering competing phases from the ICSD. However, to be self-consistent, the newly predicted stable structures should also be considered as competing phases. Therefore, we supplemented the ICSD structures with the 1707 fully relaxed structures as well as the lowest predicted energy for each of the ∼220 000 compositions in our decoration space. Of these 1707 compositions, 31 are found in the ICSD and therefore, are not considered further in our analysis. We reconstructed the convex hulls with this combined data set and calculated what we term “self-consistent (SC) decomposition energy”. After this re-evaluation, 285 structures had a SC *E*_decomp_ < 0 eV/atom. We provide the structures, predicted, volume relaxed, and fully relaxed total energies, as well as the SC decomposition energies (see Section 4.5).

### 2.5 Novel Stable Structures

Thermodynamic stability is a prerequisite in the search for novel functional materials. Beyond stability, such materials must also exhibit specific functional properties. Our motivation for this study is to find new battery materials, which inspired our choice of chemistries to build the training data set. We evaluate the suitability of the 285 newly predicted compositions/structures as solid-electrolytes in SSBs.^10^ For application in metal-anode SSBs, the solid electrolyte should have a low reduction potential, *i.e*., close to 0 V w.r.t. Li/Li^+^. For compatibility with high-voltage cathode, the oxidation potential should be large, ideally > 4.0 V. In addition, a large electrochemical stability window (ESW) is desired. The volume fraction of the structure available to the conduction ions is a rough measure of the ionic conductivity, although more refined descriptors have been proposed.^24,25^ In summary, we sought structures with the following features: (F1) SC decomposition energy < −0.1 eV/atom, (F2) low reduction potential < 2.0 V w.r.t. Li/Li^+^, (F3) high oxidation potential > 4.0 V w.r.t. Li/Li^+^, (F4) large ESW > 2.0 V, and (F5) large volume fraction available to conduction ion ≥ 30%. These criteria (F1–F5) represent a set of choices that can be easily adjusted for further analysis. Here, ESW is calculated as the difference between the oxidation and reduction potentials, which depend only on thermodynamic stability. While ESW calculated in this manner provides a useful guide, recent studies have highlighted the need to consider electronic band alignment between the electrolyte and electrodes to rigorously determine ESW.^26^ These band alignment calculations are computationally intensive and beyond the scope of our study.

**Figure 5:**
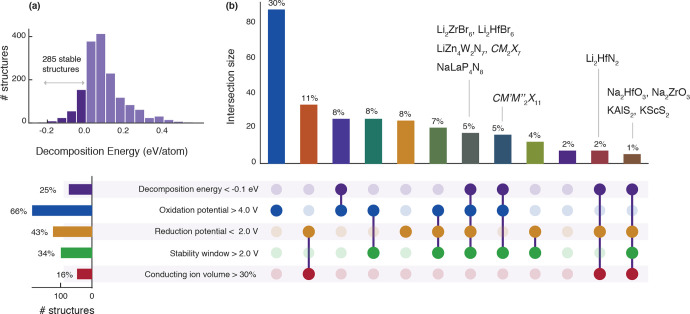
Functional features of the predicted stable structures relevant for battery applications. (a) Histogram showing the distribution of self-consistent decomposition energies for the 2003 structures originally predicted to be stable. (b) UpSet plot of the 285 candidate structures. Combinations of feature cutoffs with less than five members are not visualized. Example compositions are listed for several sets.

**Figure 6:**
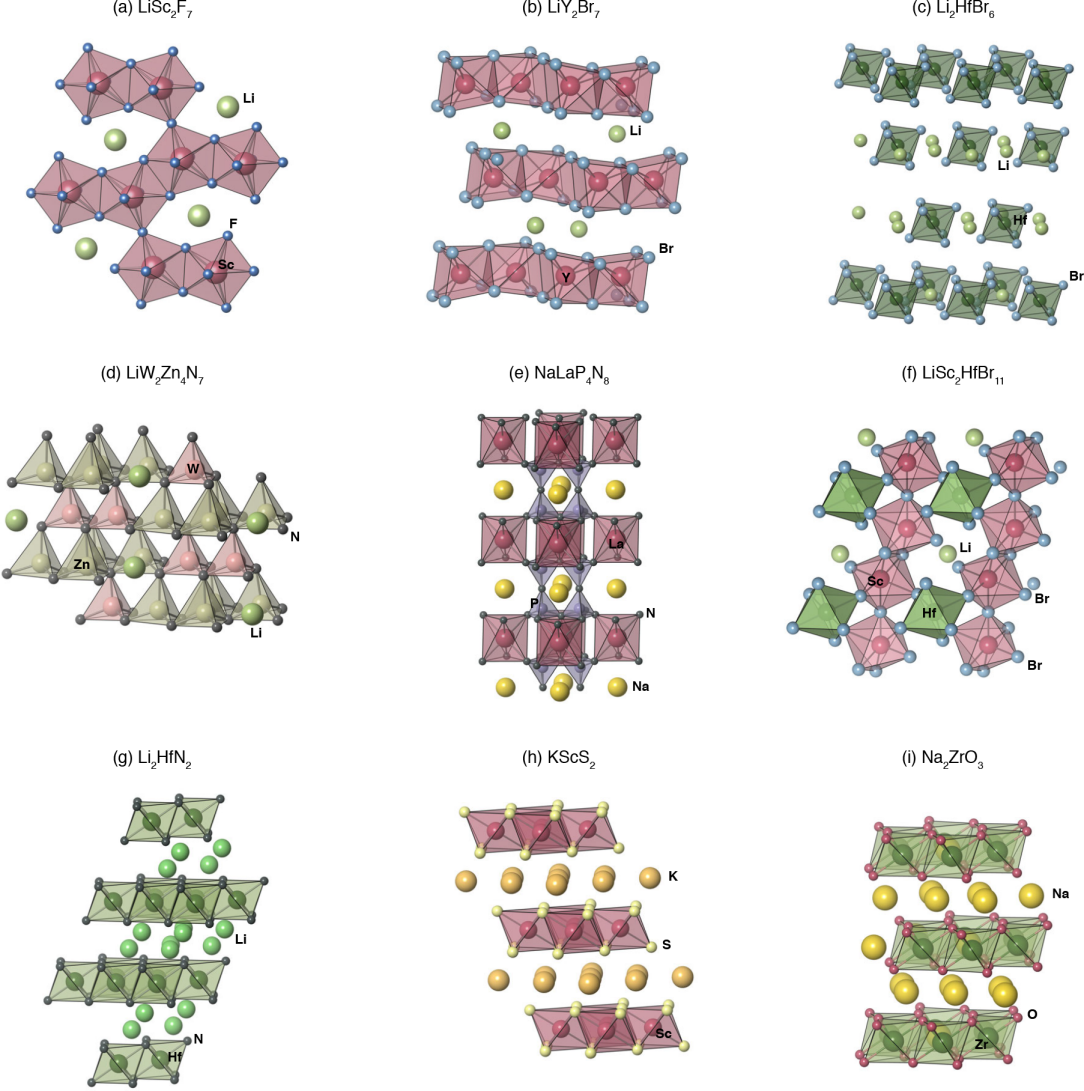
Example crystal structures predicted to be stable and exhibit certain features (Figure 5) suitable for application as solid electrolytes. (a) LiSc_2_F_7_. (b) LiY_2_Br_7_. (c) Li_2_HfBr_6_. (d) LiW_2_Zn_4_N_7_. (e) NaLaP_4_N_8_. (f) LiSc_2_HfBr_11_. (g) Li_2_HfN_2_. (h) KScS_2_. (i) Na_2_ZrO_3_.

Figure 5b shows the number of structures that pass each feature cutoff, as well as combinations of feature cutoffs. Structures that pass all cutoffs would be of particular interest. While we did not find any such structures that pass all feature cutoffs, several structures passed 3–4 cutoffs, as shown in Figure 5b. Some of these structures and family of structures are labeled in Figure 5b and their DFT-relaxed crystal structures are shown in Figure 6. As no structure simultaneously possessed all the desired features, we examined the Pareto surface of all five features. We identified 61 structures lying on the Pareto frontier, which are included in the supplemental data (see Data Availability Statement). The most interesting Pareto front occurred between conducting ion volume and the electronic stability window, which we have included as Figure S1. Here, structures with a high conducting ion volume seem to have a lower ESW and vice versa, possibly indicating a trade-off between battery lifetime (*i.e.*, stability) and performance (charge transfer rates). Six structures lie on this Pareto curve, four of which we discuss below.

A family of compounds with the general formulas *CM*_2_*X*_7_ (*C* = Li, Na; M = Sc, Y; X = halogens) are identified as stable structures that exhibit features F1 through F4, but are *C*-poor compositions, which contributes to their low conducting ion volume fraction. LiSc_2_F_7_ (Figure 6a, space group *P2*) and LiY_2_Br_7_ (Figure 6b, space group *Pnma*) are two examples from the *CM*_2_*X*_7_ family of compounds. These structures are derived from the K and In rare-earth phyllochlorides,^27^ which feature unique 7-fold coordinated trivalent rare-earths. In the DFT-relaxed structures of LiSc_2_F_7_ and LiY_2_Br_7_, shown in Figure 6, Sc and Y form edge- and corner-shared [ScF_7_] and [YBr_7_] polyhedra.

Li_2_HfBr_6_ (Figure 6c) and Li_2_ZrBr_6_ also pass the feature F1–F4 cutoffs. The predicted stable structures (space group *R*3̅) contain isolated [HfBr_6_] and [ZrBr_6_] octahedra with interspersed Li. LiW_2_Zn_4_N_7_ (Figure 6d, space group *C*2) forms a tetrahedrally bonded structure consisting of edge-connected [ZnN_4_] and [WN_4_] tetrahedra. The structure is derived from Cu_4_NiSi_2_S_7_, which crystallizes in monoclinic distorted sphalerite superlattice.^28^ NaLaP_4_N_8_ is yet another structure that fulfills F1–F4 cutoffs. The initial structure of NaLaP_4_N_8_ is created by decorating the BaSrFe_4_O_8_ trigonal (space group *P*3̅1*m*) structure,^29^ with P occupying the tetrahedrally bonded Fe sites and La on the octahedrally coordinated Sr sites.

LiHfSc_2_Br_11_ (Figure 6f) satisfies feature cutoffs F1, F2, and F4 and is derived from the NaZnZr_2_F_11_ structure (space group *C*2*m*). NaZnZr_2_F_11_ is a known stable compound that has been experimentally realized and contains octahedrally coordinated Zn, which is uncommon. [ScBr_4_] octahedra are highly distorted while [HfBr_4_] octahedra are less so. Li_2_HfN_2_ has a layered structure consisting of face-sharing [HfN_6_] octahedra interspersed with Li (Figure 6g). Interestingly, Li_2_HfN_2_ is predicted to be stable at the interface with Li-metal anode (reduction potential 0.0 V), but stable only up to an oxidation potential of 1.2 V. It also has a high conducting ion volume fraction (0.41). Together, these features make Li_2_HfN_2_ a promising candidate for Li-anode coatings. In fact, Li_2_HfN_2_ is a hypothetical structure that is predicted to be stable in the Materials Project database^30^ and has been previously proposed as a candidate material for Li-anode coatings.^31^

KScS_2_ (Figure 6h) and KAlS_2_ pass feature cutoffs F1 and F3–F5 and adopt the α-NaFeO_2_ structure (space group *R*3̅*m*). KScS_2_ is thermodynamically stable with K-metal anode (reduction potential 0.0 V) and has an oxidation potential of 2.7 V. The layered structure of KScS_2_ lends itself to a high K ion volume fraction of 0.31 and possibly facile K^+^ ion diffusion. The layered Na_2_ZrO_3_ (Figure 6i) and Na_2_HfO_3_ also fulfill F1 and F3–F5 and possess the Li_2_SnO_3_-type structure (space group *C*2/*c*). Due to its layered structure, Li_2_SnO_3_ has been studied as a promising cathode material.^32^ In fact, Na_2_ZrO_3_ is predicted to be a stable structure in the Materials Project database^30^ and Y-doped Na_2_ZrO_3_ has been theoretically investigated as a Na-rich cathode material.^33^ We predict that Na_2_ZrO_3_ should be stable against Na-metal anode, which is also confirmed by a phase stability analysis of the ternary Na–Zr–O chemical space on Materials Project.

Overall, we find that many of the 285 structures that are predicted to be stable contain group-3 (Sc, Y, La) and group-4 (Zr, Hf) elements. Most of the structures are halides, but we also find some oxides, chalcogenides, nitrides, and mixed-anion chemistries among the stable structures. The dominance of halides can be attributed to the ionic nature of the compounds containing alkali elements (Li, Na, K) and halogens—a direct consequence of the large electronegativity differences between them. Valence-balanced ionic compounds tend to have high formation enthalpies and therefore, are generally stable. Furthermore, we observe that the cations in the predicted structures adopt their preferred coordination with anions, *e.g.*, Sc, Y, La in 6-fold coordination and 4-fold coordinated Zn in tetrahedral geometry.

### 2.6 Reinforcement Learning Optimization of Structures

Although in this study we were able to predict the stability of all 14.3 million decorated structures, other structure searches where a brute-force computation would be intractable require a more efficient approach. Examples include (i) cases where the prototype and composition libraries are much larger, leading to an explosion of potential decorated structures, (ii) a costlier evaluation function, and (iii) allowing decorated structures to go “off-prototype,” meaning structure parameters (e.g., cell shape, atomic positions) are allowed to change, leading to a potentially infinite search space. Here we demonstrate the use of reinforcement learning (RL) to improve the search efficiency in such applications.

RL, particularly methods based on a directed tree search such as Monte Carlo Tree Search (MCTS), enable precise control over the search space and function(s) to optimize. MCTS has previously been demonstrated to solve complex optimization problems on both organic^34,35^ and inorganic materials.^36^ We developed an action space for the crystal structure design problem based on the steps for generating a decorated structure through ionic substitution (see the Supporting Information). We then implemented an MCTS optimization framework to find structures with desired properties, similar to the implementation by Sowndarya et al. for designing organic molecules.^35,37^ Following the approach of AlphaZero, this MCTS framework is augmented with a policy model that replaces the simulation phase (using a random policy) of MCTS with a predicted value score.

As AlphaZero was originally designed for competitive games, we used a ranked reward strategy to enable *tabula rasa* self-play for the single-player combinatorial optimization problem.^38^ In this strategy, the final reward of a rollout is rescaled to 0 or 1 depending on whether the reward is greater than the 90th percentile of the last 500 results. Thus, starting from an initially random walk over structure search space, the rollouts are guided by the policy to higher-reward structures.

**Figure 7:**
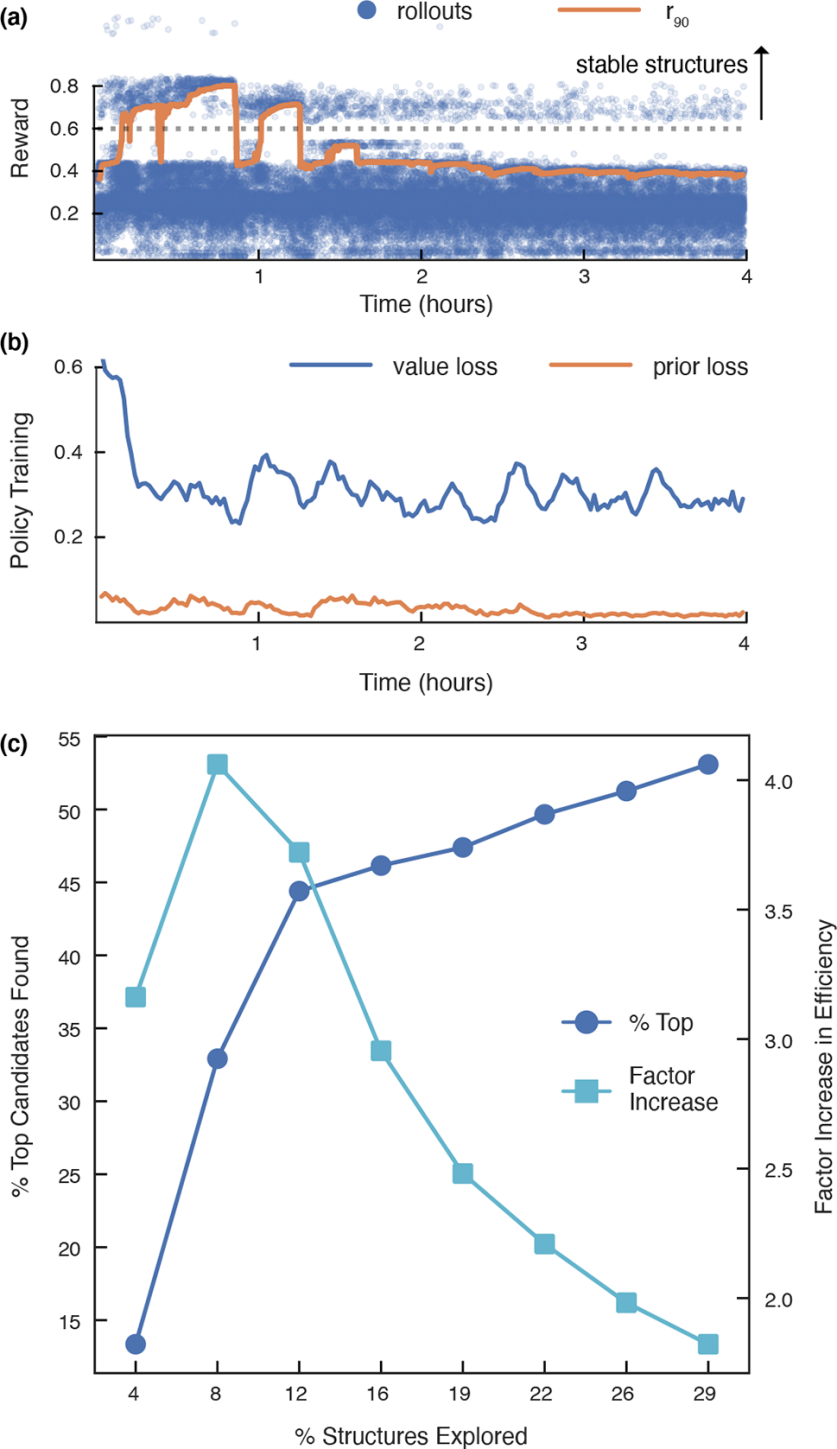
Reinforcement learning (RL) structure optimization. (a) Crystal building rollout rewards and (b) losses for the policy model vs time as the optimization proceeds. In (a), the *r*_90_ line in orange represents the cutoff at which the result was considered a win or loss. (c) Improvement in efficiency of RL compared to a brute-force search. Top candidates are structures with a decomposition energy < −0.1 eV/atom.

To search for optimal candidate structures, we also implemented a weighted reward function based on the desired features discussed in Section 2.5 (see the Supporting Information). We employed 90 rollout workers split across 5 CPU nodes for 4 h, with a single node equipped with dual Tesla V100 GPUs handling the continual training of the policy model. To improve the efficiency of the search, we continuously restricted the action space to structures not yet evaluated by any of the rollout workers. This resulted in 38 000 rollouts and ∼4.2 million structures evaluated (see Figure 7a and b for the rollout rewards and policy training losses).

We examined the improvement in efficiency for finding top candidate structures (i.e., predicted decomposition energy < −0.1) relative to the number of structures explored during the search. The largest improvement in efficiency was achieved during the beginning of the search; 34% of top-candidates were found after exploring 8% of decorations—a 4× increase relative to a brute-force search (Figure 7c). The reason the RL agent is unable to continually improve the reward and search efficiency may be because it exhausts branches of the search tree that are more densely populated with high-reward structures and is forced into continual exploration through generally low-reward spaces.

## 3 Conclusions

In this study, we have demonstrated an approach to finding stable, functional inorganic crystal structures by combining a GNN surrogate model with RL-based structural search. These results confirm the success of our upper-bound minimization strategy, where starting from a low-energy but unrelaxed starting point yields even lower energy stable crystal structures. We therefore rely on upper-bound optimization to find low-energy structure inputs, sidestepping the difficult problem of mapping unrelaxed structures to their corresponding fully relaxed energies. We demonstrate the method by searching for candidates for solid electrolytes, a demanding application that requires materials to simultaneously satisfy several competing criteria. Our method reveals 285 novel structures, many of which appear reasonable when compared with structures currently being explored for this application. In an independent but related effort, Chen and Ong predicted the stability of 31 million hypothetical structures using the M3gnet framework.^39^ Of our 285 DFT-confirmed compositions, 102 appear in the matterverse.ai web platform, with 47 of these predicted to have a negative decomposition energy. This agreement between methods with separate training data and computational approaches lends further evidence to the potential stability of these materials.

While the method presented here was demonstrated by searching over a discrete space defined by the available structural prototypes, combining our upper-bound optimization with more flexible generative methods^40,41^ is likely to result in even more stable candidate structures. Even with our current element substitution approach, by continuing to feed fully relaxed structures back to the RL agent as possible new prototypes to be decorated, we continually expand the search space considered by the optimization. Future work will also expand the number of variables optimized by the RL algorithm, targeting the search toward functional battery materials.

## 4 Computational Methods

We start with an overview of the approach, then provide specific details. To find stable, functional structures, we first generate a pool of candidate structures from a set of prototype structures and a set of suitable compositions, second, train a GNN model to predict the total energy of each of these structures, and third, calculate their thermodynamic stability w.r.t. competing phases. An implicit assumption is that some of the structures in the pool are in a stable configuration already, and our approach is simply to identify them. For our SSB application, we calculate additional features and filter the stable structures identified by the model to those with features of interest.

### 4.1 GNN Architecture

We utilized a similar GNN architecture as was developed by Pandey et al.^13^ To input crystal structures to the model, each structure is converted into a graph where each atom site is a node and the 12 nearest sites of each atom in terms of raw distances (taking periodicity into consideration) constitute the edges. For node features, we use only the identity of the elements at each site, and for edges, we use the distance (in Å) between the two sites. We use six message passing layers in the GNN. One important difference from the GNN used by Pandey et al. is that we scale each structure such that the minimum distance between atoms is 1 Å, as was done in Pal et al.^18^ Thus, the model learns a scale-invariant version of the structures.

### 4.2 Data Sets

Here we describe the prototype structures, the battery compositions, and how we decorated the prototype structures.

#### 4.2.1 Prototype Structures from ICSD

Inspired by prior studies on ionic substitution,^21,42,43^ we constructed a library of prototype structures from the ICSD by first classifying them into composition types. Here, composition type is defined as the sorted stoichiometry that is agnostic of the element type. For example, structures with compositions *A*_3_*B*_1_*C*_2_, *A*_1_*B*_2_*C*_3_, and *A*_1_*B*_3_*C*_2_ are categorized into the composition type “1–2–3”. The prototypes are limited to ordered (fully occupied lattice sites) and stoichiometric ICSD structures. At this stage, we also filtered out erroneous ICSD structures with multiple atoms occupying the same lattice site in a way that the total site occupation is larger than 1. The structures within each composition type were then deduplicated by comparing their space groups and sorted list of Wyckoff site labels. With this procedure, we constructed a prototype library containing 4000 composition types spanning > 13 000 structures.

#### 4.2.2 Battery Compositions

Most well-known solid-state battery materials, including solid electrolytes and electrodes, are ternary and multinary compounds with a distinct conducting ion (*C*), and a structural framework composed of cations (*F*) and anions (*A*). For example, the solid-electrolyte Li_3_ScCl_6_ structure contains Li^+^ ions interspersed within a framework composed of [ScCl_6_] octahedra. Therefore, we chose compositions of the general form *C*_*x*_*F*_*y*_*A*_*z*_, where *x*, *y*, *z* are the number of *C*, *F*, and *A* per formula unit. For computational tractability, we limit the search to ternary, quaternary, and quinary compositions such that *x* + *y* + *z* ≤ 15. To summarize, each composition is composed of 3–5 elements: (1) one conducting ion, *C*, (2) 1–2 framework cations, *F*, and (3) 1–2 anions, *A*. Informed by common battery chemistries, the following elements and their oxidation states are chosen: (1) **C** = Li^+^, Na^+^, K^+^, (2) **F** = Sc^3+^, Y^3+^, La^3+^, Ti^4+^, Zr^4+^, Hf^4+^, W^6+^, Zn^2+^, Cd^2+^, Hg^2+^, B^3+^, Al^3+^, Si^4+^, Ge^4+^, Sn^4+^, P^5+^, Sb^5+^, and (3) **A** = F^–^, Cl^–^, Br^–^, I^–^, O^2–^, S^2–^, N^3–^, P^3–^. We only consider valence-balanced compositions in our search. In total, there are 220 824 valence-balanced compositions spanning 174 composition types.

#### 4.2.3 Decorated Structures

For each composition, we generated decorated structures by considering all prototypes for the corresponding composition type (Section 4.2.1). For each prototype, we perform all possible decorations using ionic substitution where the elements in the prototype structure are replaced with the elements from the battery compositions (Section 4.2.2). A typical approach to generate new structures from prototypes is to establish a set of substitution rules where elements in the prototype are replaced with similar elements. In our application, we sought to remove potential human biases in the substitution process and instead constrained our substitutions solely based on valence-balanced stoichiometries. While this approach generates some structures that are very unstable and high in energy, these structures help the trained GNN to differentiate between high-energy and low-energy decorations during high-throughput screening.

For example, if the battery composition is Mg_2_ZrO_4_ and the chosen prototype is BaAl_2_S_4_ (ICSD # 35136, space group *Pa*3̅), we substitute Al with Mg, Ba with Zr, and S with O in the structure. To keep the decoration space tractable, we do not perform decorations by Wyckoff sites and simply decorate all Wyckoff sites associated with an element with the substituting element. We also consider all possible stoichiometric combinations when performing the decorations. For example, the “1–2–4” composition type allows only one unique decoration while the “1–1–1–1–1” composition type accommodates 120 unique stoichiometric decorations on the same prototype structure. We also limit the search to prototypes with less than 50 atoms in the unit cell. We note that although *C*_*x*_*F*_*y*_*A*_*z*_ compositions are inspired by the structure of well-known battery materials, we do not explicitly impose any bonding constraints (*e.g.*, *F* bonded to *A*) in constructing the decorated structures. In total, the 220 824 valence-balanced compositions result in 14.3 million hypothetical decorated structures.

### 4.3 Training Data Set

We train a GNN model to predict the total energy of a given structure, which acts as a surrogate model for DFT volume-only relaxations. To train the GNN model, we selected a subset of hypothetical decorated structures for DFT relaxation to build a training data set. Here, we describe the training data sets comprising ICSD and hypothetical structures, and the two types of DFT relaxations performed on the hypothetical structures.

#### 4.3.1 ICSD Structures

We used the same data set of ICSD structures and their total energies as was used by Pandey et al.,^13^ which consists of ∼14 000 structures available in the NREL Materials Database (NRELMatDB)^23,44^ as well as ∼2500 structures for which additional DFT calculations were performed.

#### 4.3.2 Decorated Structures for DFT Relaxation

We sampled a subset of the decorated structures (Section 4.2.3) to perform DFT calculations. For computational tractability, we sampled ternary, quaternary, and quinary compositions such that *x* + *y* + *z* ≤ 10. With these constraints, there are 914 valence-balanced battery compositions spanning 72 composition types and 150 345 decorated structures. For each composition type, we randomly selected up to 10 compositions in way that every element accommodated by that composition type (condition of valence balance) are sampled. For each composition, we then consider all prototype structures (Section 4.2.3) in the corresponding composition type. We selected ∼68 000 structures for full DFT relaxation and volume-only relaxation (Section 4.3.3).

#### 4.3.3 DFT Relaxations

DFT relaxations for the hypothetical structures were performed with VASP.^45^ Details of the calculations are previously published in refs (13) and (44). The constrained volume relaxation was also performed with VASP,^45^ using the Atomic Simulation Environment Python package.^46^ The optimization of the scalar volume was performed in a gradient-free fashion through repeated one-shot, self-consistent DFT calculations, using the Brent method implemented in scipy. Volumes were bounded between 10 Å^3^ (to prevent negative volumes) and two times a volume predicted with the data-mined lattice scheme (DLS) as implemented in Pymatgen.^47^ A rough initial volume guess (prior to DLS volume prediction) was generated with a linear model on composition trained on ICSD structures. Structures that ran into the upper bound volume during the bounded optimization tended to be unstable, *i.e.*, the energy continues to decrease as the volume increases, and were pruned from the database. Finally, we ensured the volume-energy curve was sufficiently smooth and removed structures where the minimum energy was more than 10 meV/atom lower than the second-lowest energy on the volume-energy curve.

#### 4.3.4 Data Quality Control

A number of data quality control checks were performed to remove problematic structures and relaxations from the DFT database prior to fitting the GNN model. First, we removed calculations derived from different ICSD prototypes that relax to the same final structure upon full DFT relaxation. We observed that despite being given different initial prototypes, multiple relaxations for the same composition would occasionally converge to the same fully relaxed structure. As this complicates the accurate splitting of train and validation structures, we removed duplicated results by comparing their fingerprints after relaxation (Section 4.4). We used scikit-learn to recursively cluster all fully relaxed structures, using a cosine distance of 0.01 as the distance threshold and the maximum distance between clusters as the merging criterion. For each composition, only a single fully relaxed structure per cluster was kept (one with the lowest energy), resulting in 13 133 fewer DFT data points. Next, we removed 1391 unconstrained DFT relaxations where a lower energy was obtained from the constrained relaxation. These calculations indicated that the full DFT relaxation reached a local energy minimum.

We next removed crystals with energies and volumes well outside the expected range. We fit a robust linear model to predict total energy as a function of crystal composition using scikit-learn, and removed 1551 calculations (10 full-relaxed, 1,541 volume-relaxed) with either a residual energy less than −2 eV/atom or greater than 5 eV/atom, or a residual volume less than −20 Å^3^/atom or greater than 50 Å^3^/atom. The final data set sizes are as follows: 16 409 ICSD, 52 949 fully relaxed, and 58 669 volume-relaxed structures.

### 4.4 Structure Fingerprints and Distances

Similarity between structures was calculated using the Matminer Python package.^22^ Fingerprints for each site were calculated using a local order parameter fingerprint, and converted to a crystal-level fingerprint by taking the mean and standard deviation over each site. Notably, the fingerprint method did not consider the overall volume of the unit cell, nor the chemical identity of the element at each site. Distances between crystal structures were then calculated using the cosine distance method as implemented in scikit-learn.

### 4.5 Surrogate Model Training

Of the ∼128 000 training structures, we used stratified random sampling to hold out 1500 structures for validation and 1500 for testing. We also selected 100 compositions uniformly at random and held out their structures (1492). We trained the model with a batch size of 64 structures for 100 epochs over the training data. To optimize training, we used the AdamW algorithm with an initial learning rate of 10^–4^, decayed by ∼10^–5^ each update step. We set the weight decay to an initial value of 10^–5^, also decayed by ∼10^–5^ each update step.

Here we also provide details of the learning curve evaluation. For each of five repeats, we first held out 1500 structures using stratified sampling for testing. Then, for each of the 10 training set sizes, we subsampled the training data to that size using stratified sampling, and held out 5% of that data for validation when training the model.

## Data Availability

The code and data used to train the GNN models, the main results, and structures presented in this work are available at github.com/jlaw9/upper-bound-energy-gnn (see also doi.org/10.5281/zenodo.7089031). Code used to run the RL optimization is available at github.com/jlaw9/rl_materials.

## Supporting Information Available

Plot comparing the conducting ion volume and ESW; description of the action space and reward function for the RL optimization of structures (PDF)
