# Supplementary material for: Upper-Bound Energy Minimization to Search for Stable Functional Materials with Graph Neural Networks
Source: JACS Au. 2022 Dec 31;3(1):113–23. doi: 10.1021/jacsau.2c00540 (PMC9875372; doi:10.1021/jacsau.2c00540)
Supplement: Supplementary file 1 — au2c00540_si_001.pdf [file au2c00540_si_001.pdf]

— Supplementary Information —

Upper-Bound Energy Minimization to Search for Stable Functional Materials with Graph Neural Networks

Jeffrey N. Law,*,† Shubham Pandey,‡ Prashun Gorai,*,‡,¶ and Peter C. St. John*,†

†*Biosciences Center, National Renewable Energy Laboratory, Golden, CO 80401, USA*

‡*Department of Metallurgical and Materials Eng., Colorado School of Mines, Golden, CO 80401, USA*

¶*Materials Science Center, National Renewable Energy Laboratory, Golden, CO 80401, USA*

E-mail: jeffrey.law@nrel.gov; pgorai@mines.edu; peter.stjohn@nrel.gov

Supplementary Figures

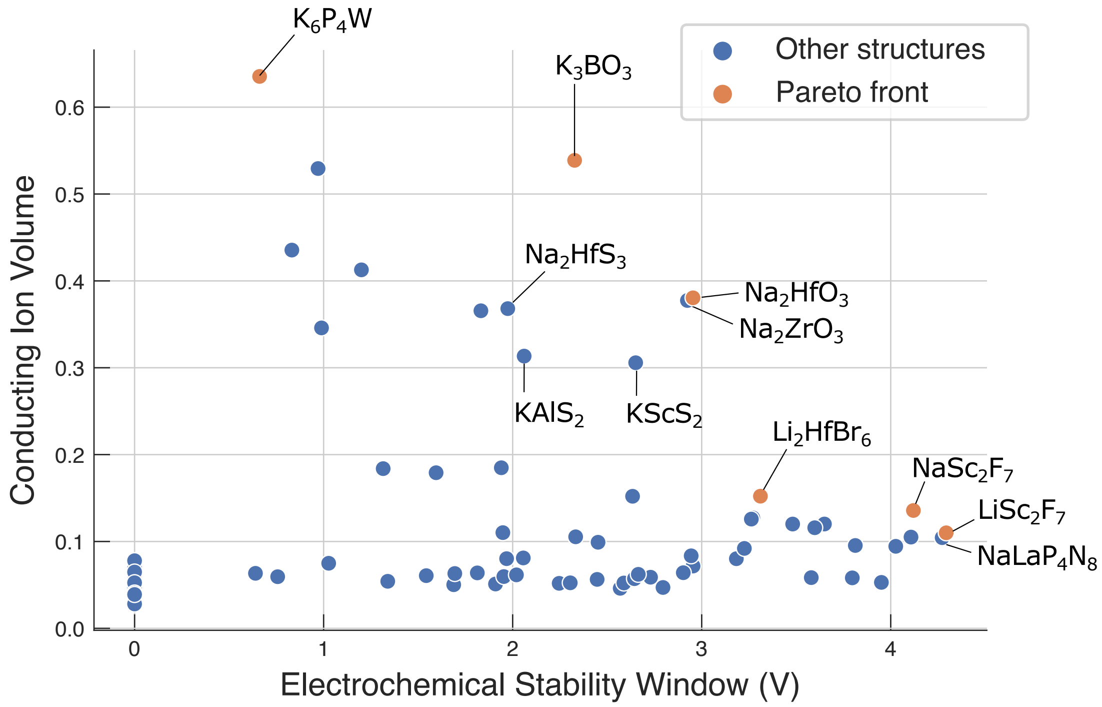

Figure S1: Scatterplot of SSB-relevant features for structures with a decomposition energy < -0.1 eV/atom.

RL Optimization of Structures for Battery Application

1. Action Space

For the RL agent to find optimal candidates, we organized the search space into six main steps (see Figure 2b). The RL agent is tasked with choosing which action to take at each step to build candidate structures with the highest reward (i.e., predicted to be stable and have other desired features).

1. *Elements.* First, one conducting ion, one to two anions, and one to two framework cations are chosen from pre-selected battery-relevant elements (Section 3.2).

2. *Composition.* The next action is to choose the stoichiometries of the elements to form a valence-balanced composition.

3. *Crystal System.* The prototype structures are grouped by crystal system (e.g., cubic). One of seven will be chosen.

4. *Decoration.* Once a prototype structure with a matching composition type is selected, the prototype's elements are replaced with the selected elements using ionic substitution. We refer to this process as decorating a structure. Depending on the composition type (i.e., element stoichiometries) of the prototype, there could be only one possible decoration (e.g., 1-2-4), or many combinations of potential element substitutions (e.g., 1-1-1-1).

5. *Energy Prediction.* The decorated structure is input to the GNN model to predict its total energy.

6. *Reward.* We then calculate the thermodynamic stability of this predicted structure by evaluating the convex hull of competing phases, where structures that lie inside the hull would decompose into other compositions. We convert this stability prediction, as well as several other battery-relevant features of the structure into a reward between 0–1.25 where larger is better (Section 2). Note that a reward > 0.6 indicates a stable structure.

We formulated steps 1–4 as two directed acyclic graphs (DAGs) as follows: the first DAG starts with a single element and ends when a battery composition is chosen (steps 1–2); the second DAG starts with the composition type, and ends when a decoration of a prototype structure is selected.

Here is an example sequence of actions taken to build the structure Na_2HfS_3 with the prototype *icsd_025019*. 1) conducting ion: Na; 2) anion: O; 3) framework cation: Hf; 4) composition: Na_2HfO_3; 5) crystal system: monoclinic; 6) prototype structure: *icsd_025019* (which has the composition ON_2Pt_3); 7) decoration: Only a single decoration is possible for the composition type 1-2-3, so O is replaced with Hf, N

by Na, and Pt by O in the structure for *icsd_025019*. At this point, there are no more actions. The reward is calculated based on the GNN's energy prediction, and this episode or game is labeled as either a win or loss depending on if the reward is larger than the rewards of 90% of the previous episodes.

2. Reward Function

We combined multiple subrewards into a single reward function using the following strategy. The subrewards are each scaled between 0 and 1, where higher is better. To control the contribution of each subreward to the final reward, we apply a weight to each subreward, listed in Table S1. If a sub-reward does not pass its cutoff (e.g., decomposition energy > -0.1 eV/atom), then we apply a penalty where we divide the weight by 2. The weights were chosen so that stable structures would always be prioritized over non-stable structures and so that the conducting ion fraction and the other three sub-rewards related to stability would contribute equally. We also give a bonus reward of 0.25 to structures that pass all cutoffs to distinguish them above the rest.

Table S1: Weights and cutoffs for sub-rewards

Sub-reward	Weight	Cutoff
Decomposition energy	2/3	-0.1 eV/atom
Conducting ion fraction	1/6	0.3
Oxidation	1/18	4.0 V
Reduction	1/18	2.0 V
Stability window	1/18	2.0 V
